# Banana bunch image and video dataset for variety classification and grading

**DOI:** 10.1016/j.dib.2025.111478

**Published:** 2025-03-18

**Authors:** D.S. Guru, Saritha N

**Affiliations:** Department of Studies in Computer Science, University of Mysore, Manasagangotri, Mysuru, Karnataka, 570006, India

**Keywords:** Banana bunch-level dataset, Banana variety classification, Banana grading, Computer vision, Machine learning, Artificial intelligence, Precision agriculture, Food science

## Abstract

Banana, a major commercial fruit crop, holds high nutritional value and widespread consumption [[4], [8],^10^]. The global banana market valued at USD 140.83 billion in 2024 is projected to reach USD 147.74 billion by 2030. Accurate variety identification and quality grading are crucial for marketing, pricing, and operational efficiency in food processing industries [9]. As wholesalers and food processing industries process bananas in bunches (not individual fruit levels) , our bunch-level dataset offers a more accurate assessment by capturing bunch-level characteristics, which are vital for grading. Existing datasets, such as [1,6], focus on individual bananas or have limited bunch-level data, highlighting the lack of large-scale bunch datasets. This dataset fills the gap by providing bunch-level images and videos of three widely consumed banana varieties-Elakki-bale, Pachbale, and Rasbale, from Mysuru, South Karnataka, India, serving as a valuable resource for food processing industries. Our dataset supports training machine learning models for bunch-level variety classification and grading of bananas and serves as a resource for research and education.

Specifications Table

**Table:** 

Subject	Computer Sciences
Specific subject area	Variety Classification and Grading of Banana at Bunch Level
Type of data	Image, Video
Data collection	The banana bunch image and video dataset were captured using an 8MP Samsung M31 and MI Note7s smartphone at random angles, producing dimensions like 2604 × 4624, 1986 × 3520, and 1080 × 1920. These dimensions are resized during preprocessing and segmented using YOLOv8.
Data source location	Banana Mandi, Devaraja Market, Shivarampet, Mysuru-570 001, Karnataka, India
Data accessibility	Repository name: Banana Bunch Image and Video Dataset Data identification number: 10.17632/dpjpdzdmsw.2 Direct URL to data: https://data.mendeley.com/datasets/dpjpdzdmsw/2 Users can download the dataset from the above URL. The Dataset is organized into folders based on variety, grade, and scale, with each image and video properly labeled. Fig. 2 illustrates the directory structure for reference and details for easy navigation.
Related research article	None

## 1 Value of the Data

• **Research Focus on Inter-Class Similarity**: The selected varieties (Elakki-bale, Rasbale, and Pachbale) exhibit high inter-class similarity in size and shape, making classification challenging. Our dataset aims to explore and address the complexity of distinguishing visually similar varieties, a vital issue in Artificial Intelligence (AI) and Machine Learning (ML) applications.
• **Classification and Grading Framework for Banana Varieties**: The dataset includes three banana varieties-Elakki-bale, Pachbale, and Rasbale-each, with three grades: Grade 1, Grade 2, and Grade 3, totalling nine classes. These nine classes enable a detailed analysis of variety-specific and grade-specific characteristics, enhancing classification and grading accuracy.
• **Scientific Relevance**: These common varieties are essential for practical applications like automated sorting and grading, benefitting food processing industries.
• **Representative and Manageable Dataset**: Limiting the study to three visually similar varieties allows us to focus on fine-grained classification tasks and detailed exploration of the AI model's performance without introducing too many varieties.
• **Foundation for Future Work**: This dataset is a foundation for future expansion, with more banana varieties to be added as needed. The current three varieties provide a sufficient challenge, especially in transfer learning and hierarchical classification approaches.
• **Model Robustness**: Including diverse backgrounds, different angles (views) , varied lighting, and distinct scales in the dataset make the machine learning models robust and predictable in real-world applications [7].
• **Integration with other agricultural datasets**: This dataset can be integrated with other agricultural datasets focusing on crop quality analysis. Future research could explore combining it with hyperspectral imaging datasets to enhance classification accuracy*.*

## 2 Background

A significant challenge in developing machine learning models for banana classification and grading is the lack of large-scale, bunch-level datasets. Current datasets focus on individual fruit, limiting model effectiveness in real-world applications. To bridge this gap, we created a comprehensive dataset of high-quality images and videos of banana bunches across various varieties and grades. Captured under diverse lighting conditions and backgrounds, this dataset enables the development of models for banana bunch detection, recognition, and classification. It also serves as a foundational resource for automated sorting and quality management, benefiting food processing industries and wholesalers. Most existing works focus on hand or finger-level classification, with limited attention to bunch-level classification. [1] achieved 93.4% accuracy in variety classification and 100% in freshness grading using CNNs on 3, 064 images, while [2] reported 93.4% and 98.3% accuracy for variety classification and quality grading, respectively, using MobileNet on 10, 000 images. However, these methods struggle with inter-class similarity and grading challenges due to limited grade categories. Our dataset, with 31, 678 images, provides a more comprehensive bunch-level dataset, addressing these limitations.

## 3 Data Description

**Fig. 1:**
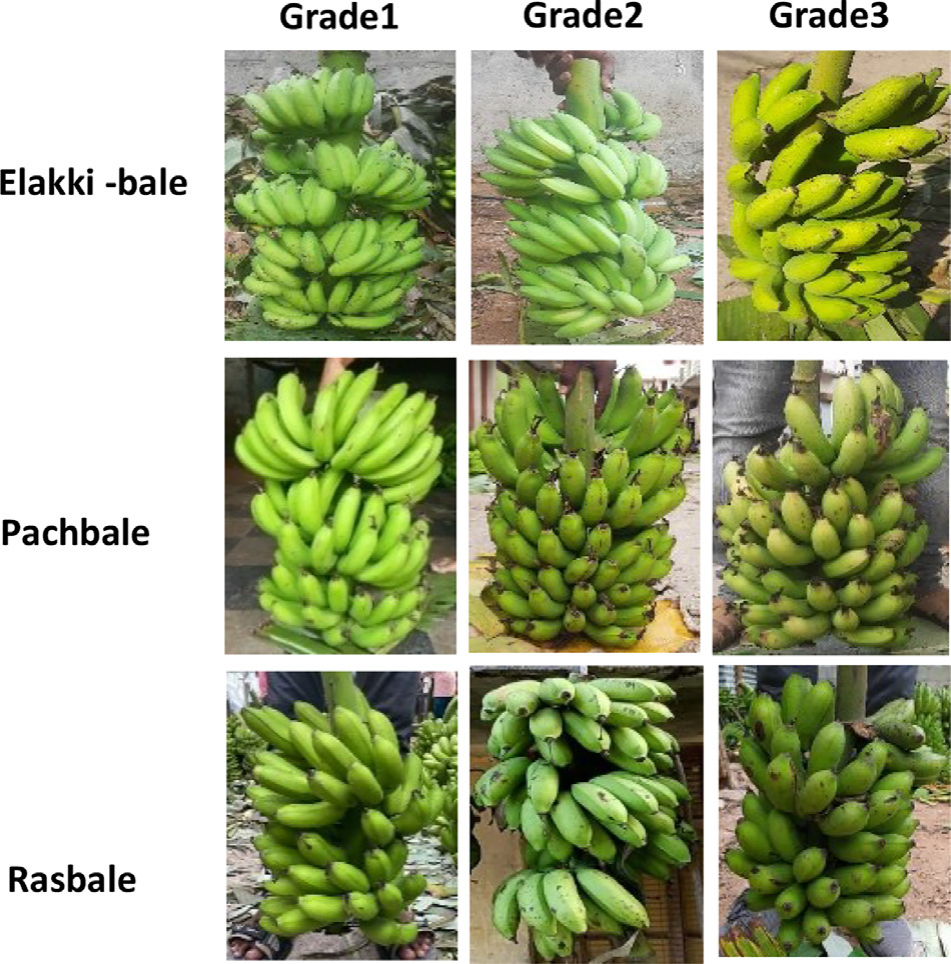
Example images of banana bunches illustrating inter-class similarity across three banana varieties and their respective grades.

**Fig 2:**
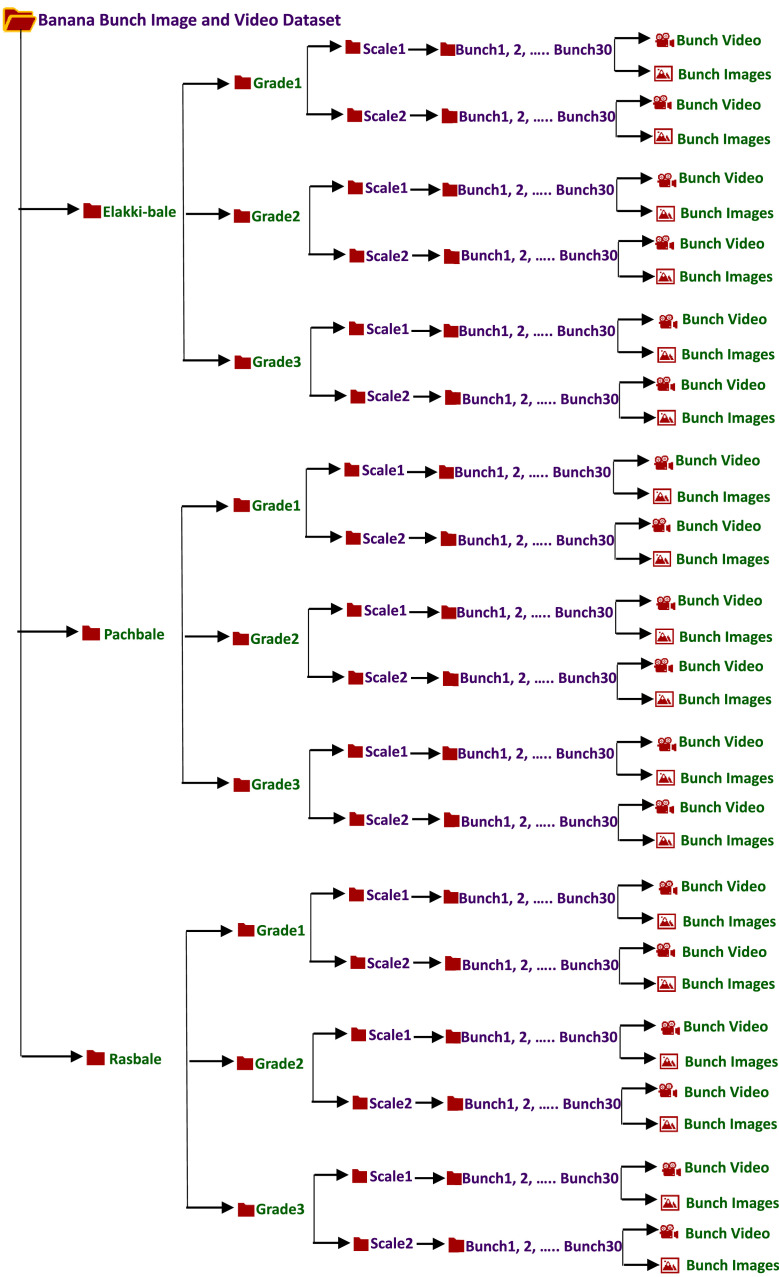
Banana bunch image and video dataset directory structure.

Our dataset comprises images and videos of three major banana varieties-Elakki-bale, Pachbale, and Rasbale cultivated in the Mysuru district of South Karnataka region, India. Each variety is categorized into three grades (Grade 1, Grade 2, Grade 3) , based on size and surface defects, resulting in nine distinct classes. Images were captured at two scales, scale 1 at a distance of 2 meters and scale 2 at 5 meters. The dataset reflects real-world market scenarios with varied lighting conditions (normal, bright and dim sunlight) and backgrounds (banana bunches, leaves, human presence). As the first reported bunch-level banana dataset, it is invaluable for training machine learning models and serves as a key resource for stakeholders like food processing industries and researchers. Fig. 1 shows sample images, and Fig. 2 outlines the directory structure of our dataset.

## 4 Experimental Design, Materials and Methods

**Fig. 3:**
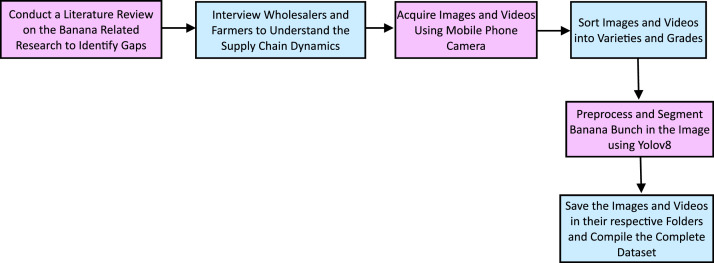
Dataset collection process.

**Fig. 4:**
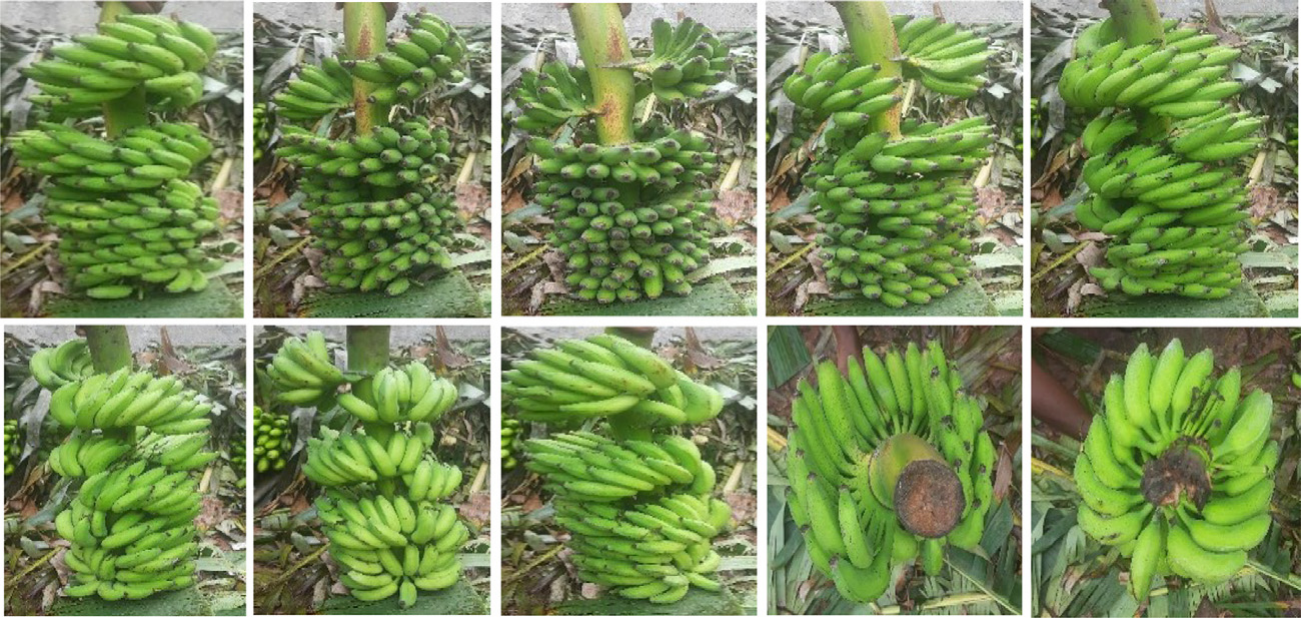
Example images of the banana bunch captured from various randomly chosen angles (views).

**Fig. 5:**
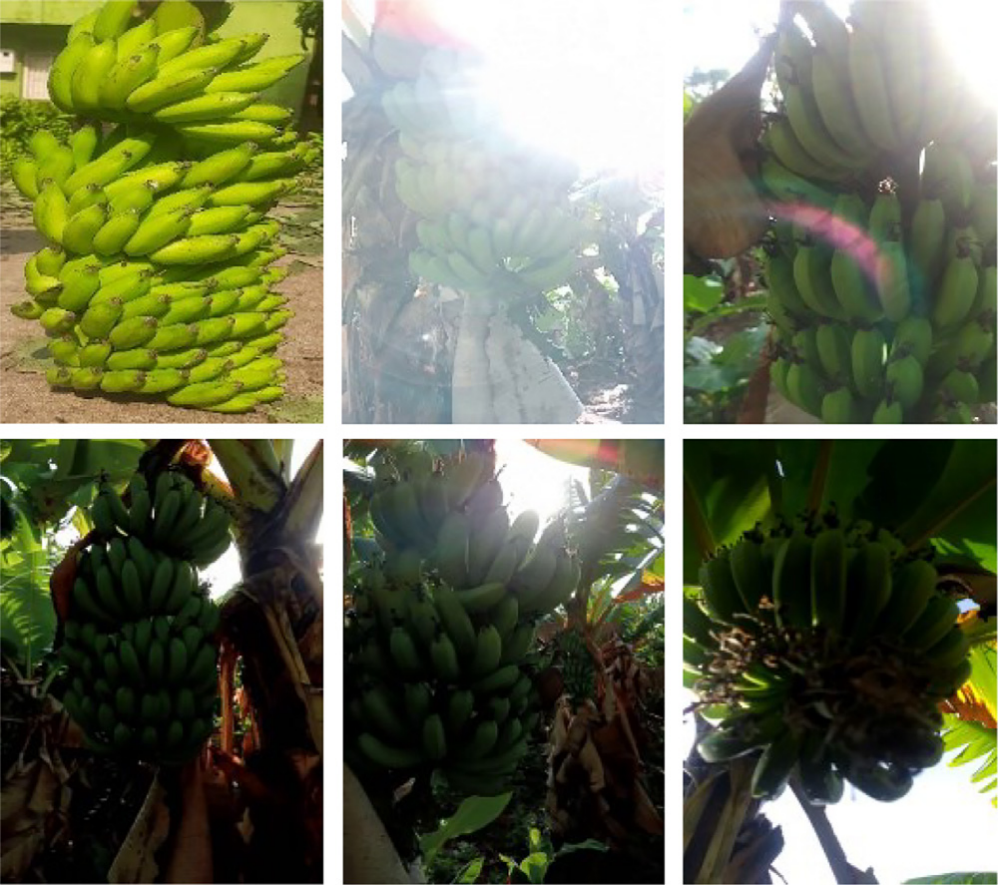
Shows images captured under normal, bright and dim sunlight.

The data collection process, shown in Fig. 3, outlines a structured process for creating a bunch-level banana dataset. It begins with a literature review to identify research gaps, followed by interviews with wholesalers and farmers to understand supply chain dynamics. Images and videos of banana bunches were captured using 8MP at random angles, producing varied dimensions resized during preprocessing. The images were sorted into varieties and grades, pre-processed, and segmented [3] using YOLOv8 before being compiled into respective folders. Fig. 4 shows sample images taken from different views, backgrounds, and lighting conditions. Variations in lighting conditions (Normal, Bright and Dim Sunlight) and angles impacted classification accuracy. Models trained on single-view images performed poorly on unseen angles, while uneven lighting caused occasional misclassifications. This highlights the need for a diverse dataset with multiple viewpoints and lighting conditions. Fig. 5 illustrate images captured under normal, bright and dim sunlight. Banana bunch images and videos were collected from the wholesale market in Mysuru, South Karnataka. The data collection process is completed in two months. Table 1 outlines the critical steps in the process.

**Table 1:** Dataset collection steps.

Sl.No.	Step	Duration	Activity
1	Dataset Collection	One month	The images of banana bunches were captured in normal, bright, and dim sunlight from different views, diverse backgrounds, and on two distinct scales.
2	Data pre-processing, segmentation and compiling of the complete dataset	One month	Images and videos were categorized by variety and grade. The sorted images are then preprocessed and segmented using a YOLOv8 pre-trained segmentation model. The dataset is compiled and stored in its respective folders.

### 4.1 Preprocessing and Segmentation

**Fig. 6:**
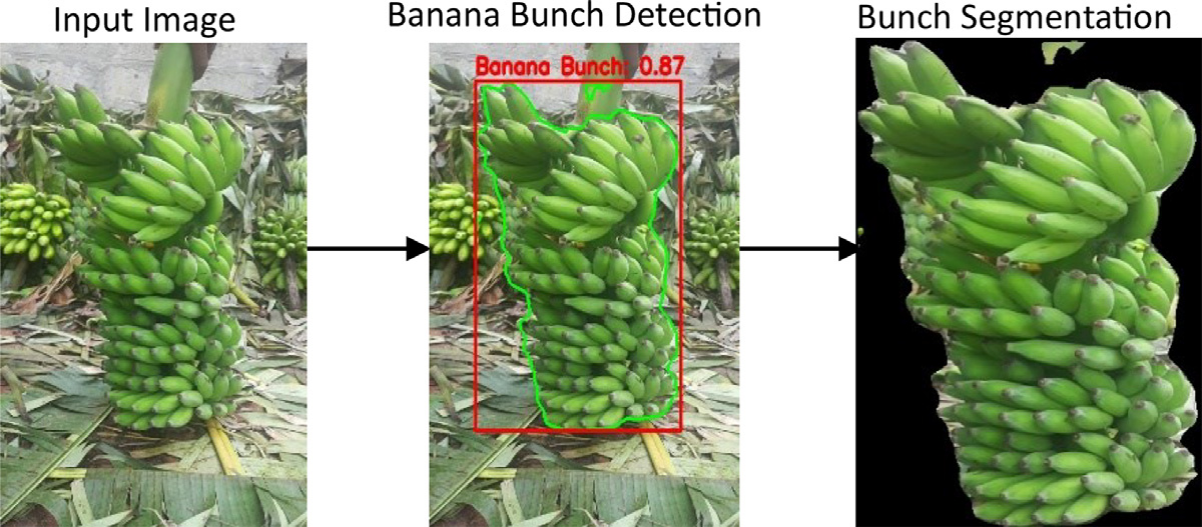
Banana bunch detection and segmentation.

**Fig. 7:**
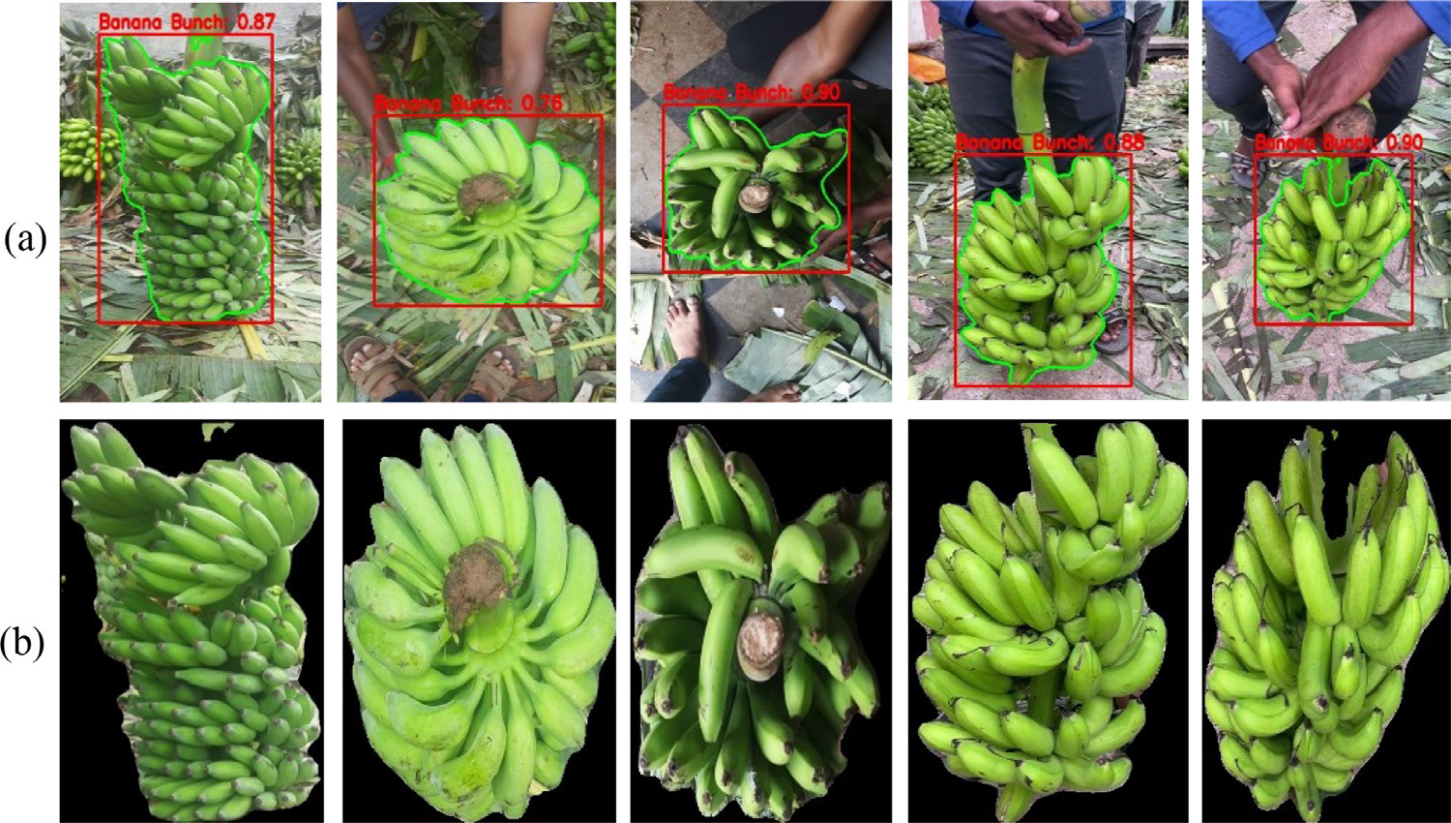
(a) Sample images with annotations for segmentation. (b) Segmented bunch images.

YOLOv8 [10], pre-trained on the COCO dataset and banana class 46 [5], was chosen for its speed, real-time processing, and efficiency in large datasets. It accurately detects, localizes, and segments banana bunches across diverse backgrounds, lighting, and scales. Unlike Mask R-CNN, which is computationally expensive, YOLOv8 balances accuracy and efficiency, making it ideal for our dataset. Fig. 6 illustrates the process. Fig. 7 (a) and (b) show the sample images with annotations for segmentation and their segmented bunch image, respectively.

Our dataset has two folders. The first folder contains videos, and the second contains segmented banana bunch images. Table 2 describes the dataset created. 547 videos and 31, 678 images were collected, categorized, and organized into folders based on their respective varieties and grades. The dataset consists of 31, 678 images and 547 videos, ensuring sufficient diversity across banana varieties, grades, lighting conditions, and scales. This dataset size is adequate for training deep learning models, enabling effective classification while preventing overfitting.

**Table 2:** Description of banana bunch image and video dataset created.

Banana Variety	Background	Sunlight	View	Grading Information	Grade	No. of Videos in each Grade	No. of Images in each Scale		No. of Images in each Scale	No. of Images in each Variety
							Scale1	Scale2		
Elakki-bale	Human beings, Human Hands, banana leaves, other banana bunches	Normal, Bright, and Dim Sunlight	Front View, Back View, Left Side View, Right Side View, Top View, Bottom View	**Grade 1 -**Large bunches, Minimal spots. **Grade 2 -**Few spots, Small variations in size. **Grade 3 -**Large number of spots, Significant variation in size	Grade1	61	1483	1533	3016	**10054**
					Grade2	61	1812	1759	3571	
					Grade3	61	1702	1765	3467	
Pachbale					Grade1	61	2009	1798	3807	**11512**
					Grade2	60	1999	1945	3944	
					Grade3	60	1917	1844	3761	
Rasbale					Grade1	62	1778	1811	3589	**10112**
					Grade2	60	1628	1687	3315	
					Grade3	61	1618	1590	3208	
Total number of images and videos in the Dataset						547	15946	15732	31678	**31678**

However, expanding the dataset by including additional banana varieties would further enhance model generalization and robustness, making it more applicable to real-world scenarios.

## 5 Results

We evaluated the dataset using a baseline MobileNetV2 model on our dataset, achieving 98.66% overall accuracy, 94.30% precision in variety identification, and 95.41% accuracy in grade classification, highlighting the dataset's effectiveness for banana variety and grading tasks.

The reported performance was achieved using a dataset with three banana varieties. However, expanding the dataset with additional varieties could impact accuracy due to increased inter-class similarity, affecting the model's real-world applicability. This necessitates further exploration of deeper architectures and advanced techniques to enhance model generalization and adaptability to real-world scenarios.

## Limitations

Due to seasonal and geographical reasons, banana availability in the market is limited, and collecting specific categories requires additional time. Some banana bunches weigh around 15 kg, making it challenging and effortful to manually hold and rotate them to capture images from various angles (views) without causing damage. Additionally, maintaining consistency while capturing images at two distant scales over an extended period is difficult.

**Challenges in Using Automated Tools:** Automated imaging systems could reduce manual effort; their implementation is impractical in real-world markets due to space constraints and continuous human activity. Fixed cameras or rotating platforms were not feasible in the wholesale market setting. Future work can explore portable camera rigs or structured data collection methods for better consistency while maintaining real-world applicability.

## Ethics Statement

The current work does not involve human subjects, animal experiments, or any data collected from social media platforms.

This dataset primarily represents banana varieties from the Mysuru region, which may introduce regional biases. Future expansions could include additional varieties from different geographical locations.

## CRediT authorship contribution statement

**D.S. Guru:** Conceptualization, Methodology, Supervision, Writing – review & editing. **Saritha N:** Software, Validation, Investigation, Data curation, Writing – original draft.

## Data Availability

Mendeley DataBanana Bunch Image and Video Dataset (Original data).
